# Real-World Experience of Bevacizumab as First-Line Treatment for Ovarian Cancer: The GINECO ENCOURAGE Cohort of 468 French Patients

**DOI:** 10.3389/fphar.2021.711813

**Published:** 2021-09-20

**Authors:** Dominique Berton, Anne Floquet, Willy Lescaut, Gabriel Baron, Marie-Christine Kaminsky, Philippe Toussaint, Rémy Largillier, Aude-Marie Savoye, Jérôme Alexandre, Catherine Delbaldo, Emmanuelle Malaurie, Hugues Barletta, Claire Bosacki, Claire Garnier-Tixidre, Philippe Follana, Hortense Laharie-Mineur, Charles Briac Levache, Bruno Valenza, Agnès Dechartres, Delphine Mollon-Grange, Frédéric Selle

**Affiliations:** ^1^ Institut de Cancérologie de l’Ouest, Saint-Herblain, France; ^2^ Institut Bergonié, Bordeaux, France; ^3^ Centre Hospitalier Princesse Grace, Monaco, Monaco; ^4^ Assistance Publique – Hôpitaux de Paris Centre-Université de Paris, Hôpital Hôtel-Dieu, Centre d’Épidémiologie Clinique, Paris, France; ^5^ Institut de Cancérologie de Lorraine, Vandœuvre-lès-Nancy, France; ^6^ Centre Léon Bérard, Lyon, France; ^7^ Centre Azuréen de Cancérologie, Mougins, France; ^8^ Institut Jean Godinot, Reims, France; ^9^ Université de Paris, Hôpital Cochin, Paris, France; ^10^ Groupe Hospitalier Diaconesses Croix Saint Simon, Paris, France; ^11^ Centre Hospitalier Intercommunal de Créteil, Créteil, France; ^12^ Centre Mistral, Guilherand-Granges, France; ^13^ Institut de Cancérologie de la Loire, Saint-Priest-en-Jarez, France; ^14^ Groupe Hospitalier Mutualiste de Grenoble, Grenoble, France; ^15^ Centre Antoine Lacassagne, Nice, France; ^16^ Clinique Tivoli, Bordeaux, France; ^17^ Clinique Francheville, Périgueux, France; ^18^ Centre Hospitalier Intercommunal de Fréjus, Saint-Raphaël, France; ^19^ Sorbonne Université, Assistance Publique – Hôpitaux de Paris, Hôpitaux Universitaires Pitié Salpêtrière – Charles Foix, Paris, France; ^20^ Centre Hospitalier Intercommunal de Cornouaille, Quimper, France

**Keywords:** bevacizumab, ovarian cancer, routine clinical practice, monitoring, progression-free survival

## Abstract

**Introduction:** Bevacizumab-containing therapy is considered a standard-of-care front-line option for stage IIIB–IV ovarian cancer based on results of randomized phase 3 trials. The multicenter non-interventional ENCOURAGE prospective cohort study assessed treatment administration and outcomes in the French real-world setting.

**Patients and Methods:** Eligible patients were aged ≥ 18 years with planned bevacizumab-containing therapy for newly diagnosed ovarian cancer. The primary objective was to assess the safety profile of front-line bevacizumab in routine clinical practice; secondary objectives were to describe patient characteristics, indications/contraindications for bevacizumab, treatment regimens and co-medications, follow-up and monitoring, progression-free survival, and treatment at recurrence. In this non-interventional study, treatment was administered as chosen by the investigator and participation in the trial had no influence on the management of the disease.

**Results:** Of 1,290 patients screened between April 2013 and February 2015, 468 were eligible. Most patients (86%) received bevacizumab 15 mg/kg every 3 weeks or equivalent, typically with carboplatin (99%) and paclitaxel (98%). The median duration of bevacizumab was 12.2 (range 0–28, interquartile range 6.9–14.9) months; 8% of patients discontinued bevacizumab because of toxicity. The most common adverse events were hypertension (38% of patients), fatigue (35%), and bleeding (32%). There were no treatment-related deaths. Most physicians (90%) reported blood pressure measurement immediately before each bevacizumab infusion and almost all (97%) reported monitoring for proteinuria before each bevacizumab infusion. Median progression-free survival was 17.4 (95% CI, 16.4–19.1) months. The 3-year overall survival rate was 62% (95% CI, 58–67%). The most commonly administered chemotherapies at recurrence were carboplatin and pegylated liposomal doxorubicin.

**Discussion:** Clinical outcomes and tolerability with bevacizumab in this real-life setting are consistent with randomized trial results, notwithstanding differences in the treated patient population and treatment schedule.

**Clinical Trial Registration:**ClinicalTrials.gov, Identifier NCT01832415.

## Introduction

In two randomized phase 3 trials (GOG-0218 and ICON7), combining bevacizumab with a carboplatin–paclitaxel doublet for newly diagnosed ovarian cancer significantly improved progression-free survival (Burger et al., 2011; Perren et al., 2011), leading to European Medicine’s Agency (EMA) regulatory approval for stage IIIB, IIIC, and IV ovarian cancer. More recently, bevacizumab-containing therapy was approved by the US Food and Drug Administration for stage III or IV ovarian cancer following initial surgical resection (Avastin Prescribing Information, 2020).

In the GOG-0218 trial, bevacizumab was associated with a significant increase in grade ≥2 hypertension versus chemotherapy alone (Burger et al., 2011). However, hypertension was generally manageable and rarely required treatment discontinuation. Bevacizumab was also associated with increased grade ≥3 proteinuria, which appears to be cumulative, and grade ≥2 gastrointestinal events, which were nevertheless relatively infrequent. Most adverse events occurred during the combination chemotherapy phase rather than during single-agent bevacizumab maintenance therapy. Similar patterns were seen in the ICON7 trial using a lower dose and shorter treatment duration of bevacizumab (Perren et al., 2011).

In France, more than 5,000 new cases of ovarian cancer are diagnosed and almost 3,500 women die from ovarian cancer each year (Institut National du Cancer (INCa), 2021). Bevacizumab was rapidly adopted into routine clinical practice in France and according to guidelines endorsed by the French National Cancer Institute (INCa), adding bevacizumab to chemotherapy should be proposed to patients with International Federation of Gynecology and Obstetrics (FIGO) stage III and IV disease, particularly in those with a poor prognosis (stage IV, residual disease after surgery, or inoperable disease) (de la Motte Rouge et al., 2019). The ENCOURAGE study aimed to collect information on treatment administration and clinical outcomes in the real-world setting from a patient population more representative of routine clinical practice than selected populations eligible for phase 3 trials. We report final results from ENCOURAGE.

## Materials and Methods

ENCOURAGE (NCT01832415) was a multicenter non-interventional single-arm prospective French cohort study led by the Groupe d’Investigateurs Nationaux pour l’Etude des Cancers de l’Ovaire et du sein (GINECO). The study was conducted in accordance with the ethical principles of the Helsinki agreement and all subsequent amendments, conformed to French legal requirements, and was approved by the Comité Consultatif sur le Traitement de l’Information en Matière de Recherche dans le Domaine de la Santé and the Commission Nationale de l’Informatique et des Libertés. All patients consented verbally to participate and the treating physician signed a form of non-objection, noting that the patient had consented to have their clinical data recorded in the framework of this non-interventional study.

The primary objective was to assess the safety profile of front-line bevacizumab therapy for ovarian cancer in routine clinical practice, focusing particularly on cardiovascular (hypertension, thromboembolism), renal (proteinuria), hemorrhagic (bleeding), gastrointestinal (gastrointestinal perforation, fistula, abscess), and post-operative effects. Secondary objectives included describing the characteristics of patients treated in routine practice, evaluating bevacizumab indications and contraindications in presenting patients, describing treatment regimens (dose, duration, and chemotherapy partner) and co-medications (particularly antihypertensives) used in routine practice, evaluating patient follow-up and monitoring (type and frequency of blood pressure monitoring, proteinuria assessment), evaluating progression-free survival (PFS) and overall survival (OS), and evaluating subsequent chemotherapy at recurrence.

Women aged ≥18 years, with a diagnosis of ovarian, fallopian tube, or primary peritoneal cancer planned for first-line bevacizumab-containing therapy were eligible. Participation in another clinical trial while participating in ENCOURAGE was prohibited.

The study was conducted in centers representative of patients presenting with ovarian cancer in routine oncology practice in France (private practice, non-academic hospitals, academic hospitals). Patients were followed for 36 months according to each participating center’s standard practice. Clinical data were recorded in an electronic case report form at baseline and at 6, 12, 18, and 36 months after initiating bevacizumab.

Data were reported using descriptive statistics [frequency (%) for categorical data and median (range or interquartile range) for quantitative data]. OS and PFS with 95% confidence intervals (CIs) and median survival with 95% CIs were estimated using Kaplan–Meier methodology. Data were analyzed using SAS Version 9.4 (SAS Institute) and R Version 3.5.1.

## Results

**FIGURE 1:**
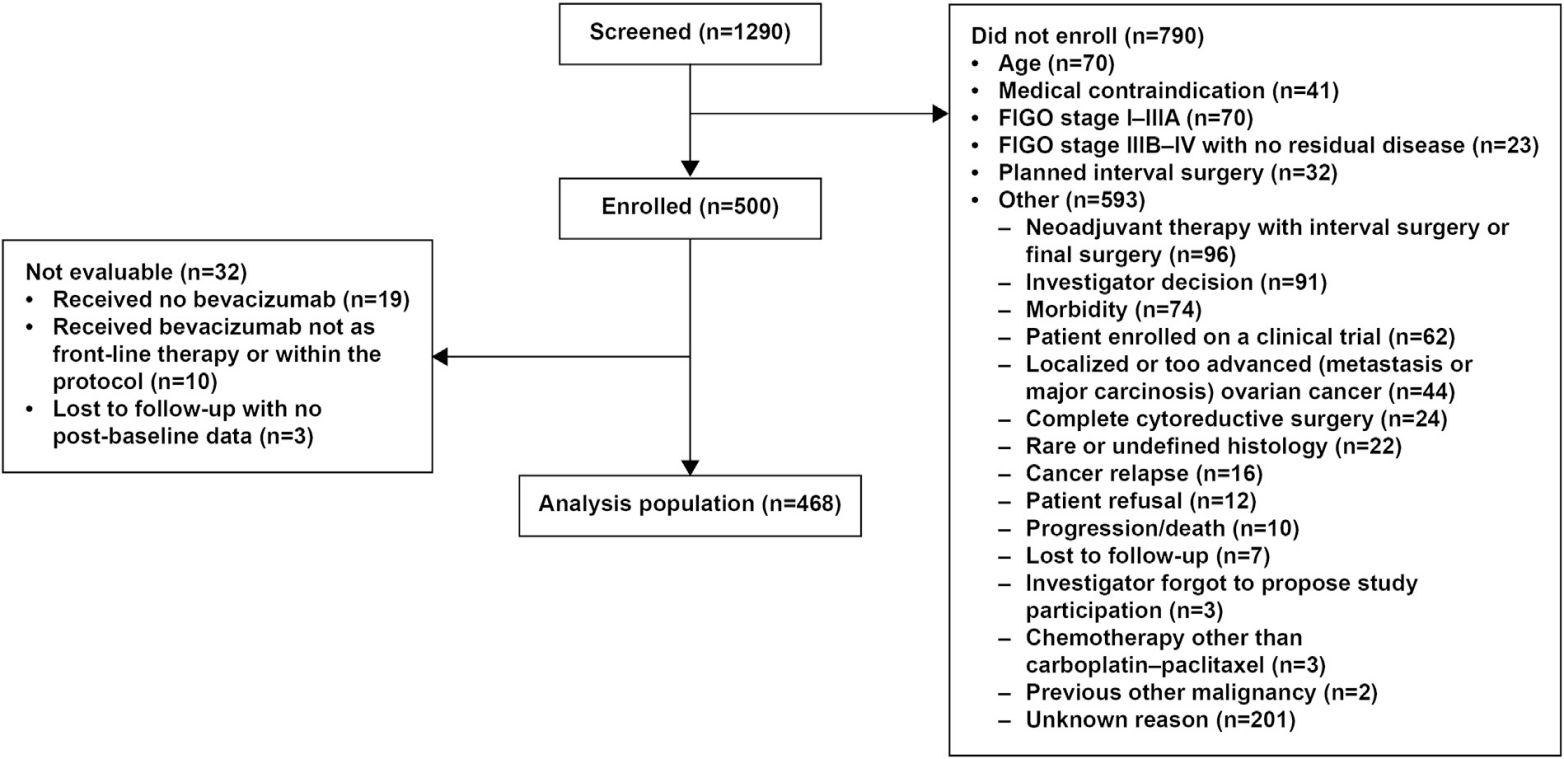
Patient flow. More than one reason for not enrolling is possible. A total of 667 “other” reasons were reported in 593 patients.

Between April 2013 and February 2015, 1,290 patients at 103 participating centers involving 205 investigators were screened. Of these, 790 patients were not enrolled for participation in this non-interventional study because of: age (*n* = 70); medical contraindication (*n* = 41); FIGO stage I–IIIA (*n* = 70); FIGO stage IIIB–IV with no residual disease (*n* = 23); planned interval surgery (*n* = 32); or other reason (*n* = 593) (more than one reason possible) (Figure 1). The most commonly reported contraindications were a history of venous thrombosis (*n* = 20) or a history of fistula, perforation, or abscess (*n* = 9). Of the 500 patients ultimately enrolled, 468 were included in the analysis and 32 were not evaluable (Figure 1).

Table 1 shows baseline characteristics. Of the 468 analyzed patients, 164 (35%) were treated in private practice, 131 (28%) in non-academic hospitals, 99 (21%) in anticancer centers, and 74 (16%) in academic hospitals. Most investigators (141 of 187 who enrolled analyzed patients; 75%) had been involved in clinical trials within the preceding 3 years and 117 (63%) participated in gynecology societies.

**TABLE 1:** Baseline characteristics of analyzed patients (*N* = 468).

Characteristic	No. of patients (%)
Median age, years (range) [interquartile range]	64.8 (23–90) [58.1–70.5]
ECOG performance status	
0	155 (33)
1	218 (47)
2	19 (4)
3	1 (<1)
Not done	75 (16)
Pre-existing conditions at study entry^a^	148 (32)
Arterial hypertension	130 (28)
Type 2 diabetes	20 (4)
BMI >30 kg/m^2^	30 (6)
Ongoing treatment at study entry^a^	177 (38)
Antihypertensive therapy	124 (26)
Statin	41 (9)
Anticoagulant therapy	46 (10)
FIGO stage at diagnosis	
I	6 (1)
II	5 (1)
IIIA	5 (1)
IIIB–IV	418 (89)
Unknown	34 (7)
Histology	
Serous	387 (83)
Endometrioid	36 (8)
Clear cell	7 (1)
Mucinous	7 (1)
Other	31 (7)
Primary surgery including laparoscopy for staging	427 (91)
Complete debulking	125 (27)
Interval debulking surgery before starting bevacizumab	220 (47)
Complete interval debulking	156/464 (34)
*BRCA* mutation status	
Mutant	38 (8)
*BRCA1* ^a^	24 (5)
*BRCA2* ^a^	16 (3)
Wild type	109 (23)
Unknown	321 (69)
Baseline urine dipstick result	(*n* = 341)
0	292 (62)
Trace	25 (5)
1+	18 (4)
2+	6 (1)
24-h protein measurement	13/467 (3)
Median (range) [interquartile range], g/24 h (*n* = 13)	0.1 (0–1) [0.1–0.2]

^a^ More than one answer possible.

BMI, body mass index; ECOG, Eastern Cooperative Oncology Group; FIGO, International Federation of Gynecology and Obstetrics.

Most patients (405 of 468; 87%) received bevacizumab at a dose of 15 mg/kg every 3 weeks or equivalent. Of the remainder, 38 (8%) received bevacizumab at a dose of 7.5 mg/kg every 3 weeks (as used in ICON7) and 25 (5%) received another schedule. Bevacizumab was combined with carboplatin in 464 patients (99%) and paclitaxel in 458 patients (98%) [other chemotherapy partners included gemcitabine in four patients (0.9%), oxaliplatin and docetaxel each in two patients (0.4%), and cyclophosphamide in one patient (0.2%)]. By the data cut-off date, all but four patients had discontinued bevacizumab. The median interval between initiating chemotherapy and first bevacizumab dose was 77.5 (range 0–494, interquartile range 21–171) days. The median number of bevacizumab cycles was 18 (range 1–53, interquartile range 10–21), corresponding to a median duration of 12.2 (range 0–28, interquartile range 6.9–14.9) months. Bevacizumab was continued beyond the planned 15 months in 106 patients (23%); reasons for prolonging treatment were not collected. Among the 463 patients who had discontinued bevacizumab at the data cutoff, bevacizumab treatment was discontinued because of disease progression in 169 patients (37%) and because of toxicity in 36 (8%).

**FIGURE 2:**
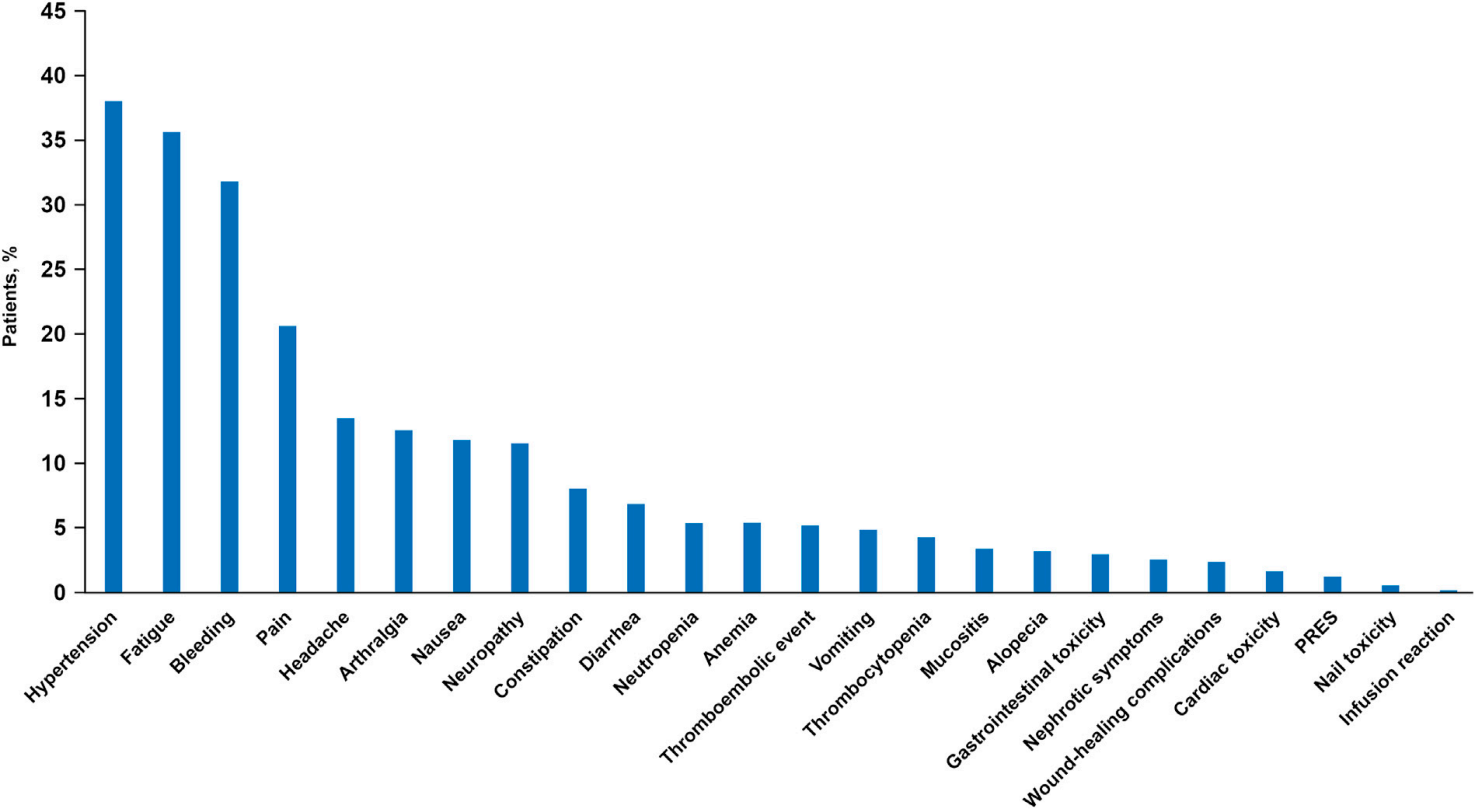
Percentage (in descending order) of patients experiencing adverse events (all grades). Verbatim terms recorded under target adverse event categories were as follows: thromboembolic events: phlebitis (*n* = 8), pulmonary embolism (*n* = 6), cerebrovascular accident (*n* = 1), other (*n* = 12). Gastrointestinal toxicity: subileus (*n* = 2), gastrointestinal perforation, constipation, abdominal distention, upper abdominal pain, dyspepsia, fistula, enterocutaneous fistula, perineal fistula, umbilical hernia, intestinal occlusion, wound evisceration/stomal complication/fistula/dehiscence/urinoma, gastrointestinal disorders (each *n* = 1). Cardiac toxicity: hypertension (*n* = 2), palpitations (*n* = 2), pulmonary artery hypertension, aortic insufficiency/palpitations/supraventricular extrasystole, acute coronary syndrome, tachycardia, poorly defined (each *n* = 1). PRES, posterior reversible encephalopathy syndrome.

The most common adverse events were hypertension (any grade in 177 patients; 38%), fatigue (166 patients; 35%), bleeding [148 patients, 32%; predominantly epistaxis (152 episodes) and gum bleeding (48 episodes)], pain (96 patients, 21%), headache (63 patients, 13%), and arthralgia (59 patients, 13%) (Figure 2). Six patients (1%) developed posterior reversible leukoencephalopathy syndrome, which led to hospitalization in one patient. Gastrointestinal perforation, fistula, and arterial events were infrequent (Figure 2). Proteinuria occurred in 25 patients (5%). There were no cases of congestive heart failure and no treatment-related deaths. Serious adverse events were reported in 17 patients (4%; 18 events), of which one event was grade 4, 13 were grade 3, three grade 2, and one of unknown grade.

Most physicians (168 of 187; 90%) reported blood pressure measurement immediately before each bevacizumab infusion; 14 (7%) reported blood pressure measurement several times between each infusion; and five (3%) reported less frequent blood pressure measurement. Table 2 provides details of hypertension detection and management. Hypertension was most frequently detected by an oncologist (58%), and 68% of episodes required initiation or modification of antihypertensive therapy. Almost all physicians (181; 97%) reported monitoring for proteinuria before each bevacizumab infusion, typically using a urine dipstick. 24-h protein monitoring was requested following a urine dipstick result of 2+ by 103 clinicians (55%) and of 3+ in 66 (35%) [1+ in 7 (4%), missing in 11 (6%)].

**TABLE 2:** Description of hypertensive episodes in patients receiving bevacizumab therapy.

Hypertension diagnosis and management	No. of episodes (%)
Healthcare professional detecting hypertensive episode^a^	
Oncologist	149 (58)
Nurse	72 (28)
General practitioner	25 (10)
Cardiologist	15 (6)
Other	27 (11)
Consultation with cardiologist	90/257 episodes (35)
Median maximal blood pressure (range) [interquartile range], mmHg	
Systolic (*n* = 229)	165 (20–220) [154–180]
Diastolic (*n* = 216)	90 (55–193) [81.5–100]
Antihypertensive therapy initiated or modified^b^	176/257 (68)
First therapy initiated	98 (38)
Additional drug administered	46 (18)
Class of antihypertensive changed	37 (14)
Dose increased	29 (11)
Type of antihypertensive drug(s)^b^	
Calcium channel blocker	114 (44)
Beta blocker	64 (25)
Angiotensin-converting enzyme inhibitor	64 (25)
Angiotensin receptor blocker	60 (23)
Thiazide diuretic	35 (14)
Other^c^	9 (4)

a A single hypertensive episode could be detected by more than one healthcare professional.

b Patient may receive more than one anti-hypertensive drug.

c Alpha-adrenoreceptor antagonist (n = 6), combination of beta blocker and thiazide diuretic (*n* = 1), loop diuretic (*n* = 1), other peripheral vasodilator (*n* = 1).

257 hypertensive episodes (any grade) reported among 177 of the 468 analyzed patients.

Overall, 165 patients (35%) underwent 234 surgical procedures after initiating bevacizumab. Surgery comprised 91 tumor resections, 45 abdominopelvic procedures to treat complications, 23 central line procedures, 13 dental procedures, and “other” procedures in the remaining 62 cases (30 of which were considered minor, such as ascites puncture, urinary catheter ablation, colonoscopy/gastroscopy). Most (116 patients, 25%) underwent only one surgical procedure but 35 patients had two procedures, nine had three procedures, four had four procedures, and one had five procedures. The median interval between last bevacizumab dose and surgery was 2.1 months (range 0–30 months, interquartile range 1.2–9.7). Secondary effects or complications were observed within 30 days following surgery in 34 (15%) of the 234 procedures, most commonly mild-to-moderate wound-healing complications (eight procedures; 3%), moderate-to-severe infection (four procedures; 2%), or moderate bleeding (four procedures; 2%). There were three cases of intestinal perforation or fistula within 30 days of surgery (one mild, two severe).

**FIGURE 3:**
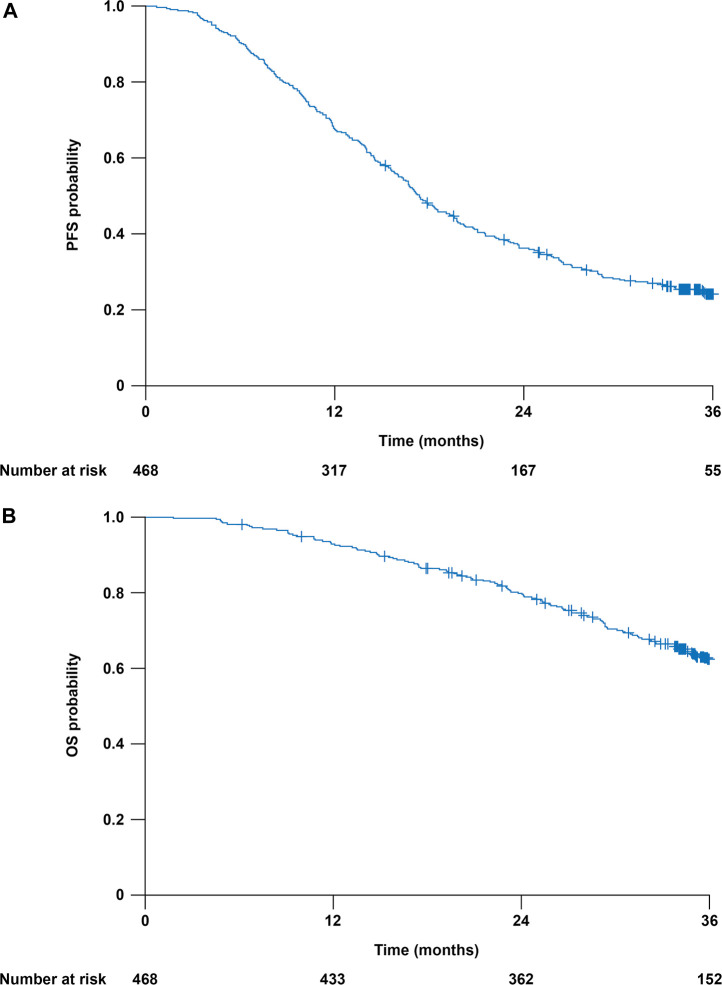
Clinical outcomes in the 468 analyzed patients. Kaplan-Meier curves are truncated at 36 months. **(A)** Progression-free survival (PFS). **(B)** Overall survival (OS).

At the data cut-off (August 23, 2018), the median follow-up duration was 35.2 months (range 1.8–44.0; interquartile range 25.4–36.3). At this date, 273 patients (58%) had completed follow-up as planned, 172 (37%) had died, 18 (4%) were lost to follow-up before completing the protocol-required 36-months follow-up, three (1%) were being followed in another hospital, and two (<1%) had discontinued because of technical/administrative reasons. At the data cut-off, 114 patients (24%) were alive without progression, 349 (75%) had experienced disease progression, and five (1%) had died without evidence of progression. Median PFS was 17.4 (95% CI 16.4–19.1) months (Figure 3A). The 1-year PFS rate was 68% (95% CI, 64–73%) and the 3-year PFS rate was 25% (95% CI, 21–29%). Overall, 172 patients (37%) had died at the date of study completion. All but 11 deaths were attributable to disease progression. The 3-year OS rate was 62% (95% CI, 58–67%) (Figure 3B).

Information on subsequent therapy was available for 327 (94%) of the 349 patients with recorded disease recurrence. The most commonly administered chemotherapy (alone or in combination) at recurrence was carboplatin (219 of 327 patients; 67%), followed by pegylated liposomal doxorubicin (181; 55%). Forty-four patients (13%) received bevacizumab therapy and 19 (6%) received olaparib.

## Discussion

In French routine practice, front-line bevacizumab is usually administered according to the EMA label. The vast majority of patients received bevacizumab 15 mg/kg every 3 weeks combined with a carboplatin–paclitaxel doublet. Clinical outcomes and tolerability in this real-life setting are consistent with reports from randomized trials, notwithstanding differences in the treated patient population and treatment schedule.

Since initiating this study, results have been published from the single-arm phase 3B ROSiA study in more than 1,000 patients from 35 countries (Oza et al., 2017). ROSiA adopted a pragmatic, real-world approach, with clinicians choosing their preferred schedule of paclitaxel (weekly or every 3 weeks) and bevacizumab (although most chose 15 mg/kg every 3 weeks). Importantly, bevacizumab could be continued for up to 24 months, or even longer if patients were still deriving clinical benefit. Until recently, the optimal bevacizumab treatment duration had been an unanswered question in the gynecologic oncology community ever since the GOG-0218 and ICON7 trials were first presented (Marth et al., 2017; Musella et al., 2017). Results from the single-arm ROSiA study hinted toward improved outcomes with longer treatment duration, but had inherent bias because perceived benefit was not assessed by imaging. In the ENCOURAGE study, most patients received treatment until disease progression and the median treatment duration was 18 cycles (interquartile range 10–21). Almost one fourth of patients received bevacizumab for longer than the planned 15 months, yet median PFS was consistent with trials evaluating 15 months of bevacizumab. Recently, definitive results on bevacizumab duration were reported from the randomized phase 3 AGO-OVAR17/BOOST trial (NCT01462890) comparing 15 *versus* 30 months of bevacizumab. Longer bevacizumab treatment duration did not improve PFS or OS, and therefore 15 months of bevacizumab remains the standard duration (Pfisterer et al., 2021).

One of the main objectives of the ENCOURAGE study was to assess tolerability, particularly the incidence and management of selected adverse events typically associated with bevacizumab-containing therapy. The 38% incidence of any-grade hypertension appears to be higher than the 26% incidence reported in ICON7 with bevacizumab 7.5 mg/kg every 3 weeks for 12 months (Perren et al., 2011) and marginally higher than the 32% incidence in GOG-0218 with bevacizumab 15 mg/kg every 3 weeks for 15 months (Avastin Prescribing Information, 2020). However, given the focus on hypertension in the ENCOURAGE case report form, increased recognition of this side effect, and the requirement to record the frequency of monitoring, increased vigilance for hypertensive events may have contributed to greater awareness and reporting. Additionally, the population was less selected in ENCOURAGE than in randomized phase 3 trials. The incidence of all-grade hypertension in ENCOURAGE appears to be lower than the 55% incidence reported in the single-arm ROSiA study, in which bevacizumab was administered for up to 36 months typically at 15 mg/kg every 3 weeks. As noted by the ROSiA investigators, hypertension may occur in later cycles and therefore the treatment duration in ENCOURAGE (longer than in ICON7, shorter than in ROSiA) may contribute to the hypertension incidence observed in ENCOURAGE (higher than in ICON7, lower than in ROSiA). Differences in the grading scale for assessing hypertension may also contribute (Oza et al., 2017; Plummer et al., 2019).

Another well-documented side effect of bevacizumab-containing therapy is epistaxis. In ENCOURAGE, the vast majority of bleeding events were epistaxis. The 32% incidence of bleeding is similar to the 39% incidence reported in ICON7 (Perren et al., 2011). Few trials report low-grade bleeding as this is generally considered a manageable event requiring minimal or no medical intervention (Miles et al., 2010). Patients may experience bleeding gums, blood-stained mucus after nose blowing, or epistaxis requiring only basic first aid (Miles et al., 2010).

Gastrointestinal perforation occurred in only one patient (0.5%), no higher than in the ICON7 trial (Perren et al., 2011) or ROSiA (Oza et al., 2017), and perhaps reflecting increased familiarity and caution surrounding this side effect. The 5% incidence of proteinuria (any grade) in ENCOURAGE is within the range reported in ICON7 (5% any grade with a shorter duration of bevacizumab) and ROSiA (4% grade ≥3 with a longer duration of bevacizumab), although in the UK OSCAR study proteinuria was reported at any grade in 20% of patients and at grade ≥3 in 1% (Hall et al., 2020). All such cross-trial comparisons should be interpreted with considerable caution given differences in study design, patient selection, treatment administration, and adverse event monitoring and follow-up.

A potential weakness of the study is the less rigorous monitoring and data collection and more heterogeneous patient population and clinical assessment typical of an observational study, such as ENCOURAGE, versus a double-blind, placebo-controlled randomized phase 3 trial. However, this is also the strength and objective of the study: to explore whether outcomes in highly selected populations can be replicated in the real-world setting in patients with more comorbidities and frailties that may prevent them enrolling into randomized clinical trials. Similar studies reporting outcomes in routine clinical practice have been reported from the UK, Germany, and Japan (Komiyama et al., 2019; Hall et al., 2020; Sehouli et al., 2020). As with ENCOURAGE, these studies showed a safety profile consistent with phase 3 reports, with no new safety signals. All-grade hypertension was reported in 32% of patients in OSCAR, 23% in JGOG3022, and 17% in OTILIA. A limitation in such comparisons is that reporting of adverse events was in accordance with local practice, and therefore subject to reporting bias. A strength of these country-specific studies compared with global studies such as ROSiA is the more homogenous population, representing healthcare systems in a single country and thus providing more relevant information on expected outcomes for oncologists in those countries to discuss with their patients. For example, in the United Kingdom OSCAR study, only 21% of patients underwent primary debulking surgery and 43% received no surgery (Hall et al., 2020), whereas in the French population treated in ENCOURAGE, 91% underwent primary surgery and 47% had interval debulking surgery, resulting in complete resection in 27% and 33% of patients, respectively.

Median PFS of 17.4 months in ENCOURAGE is within the range reported in previous prospective trials evaluating bevacizumab therapy for up to 15 months [GOG-0218, ICON7, OTILIA, OSCAR, JGOG3022: 15.4–19.4 months (Burger et al., 2011; Perren et al., 2011; Komiyama et al., 2019; Avastin Prescribing Information, 2020; Hall et al., 2020; Sehouli et al., 2020)] but shorter than the median PFS of 25.5 months reported in the ROSiA study (Oza et al., 2017). Most recently, results from the PAOLA-1 randomized phase 3 trial showed median PFS of 16.6 months with front-line bevacizumab and chemotherapy (consistent with findings in our study), but this was increased to 22.1 months with the addition of maintenance olaparib therapy (Ray-Coquard et al., 2019). Enrollment to the ENCOURAGE study was completed before polyADP ribose polymerase (PARP) inhibitors became standard-of-care therapy in the recurrent setting and before data were available in the front-line setting. One may assume that in France, many of the population treated in ENCOURAGE, particularly those with *BRCA*-mutated tumors, would now be considered for maintenance olaparib after completing chemotherapy. At recurrence, relatively few patients received bevacizumab re-treatment; based on results from the MITO16B-MaNGO OV2B-ENGOT OV17 randomized phase 3 trial (Pignata et al., 2021), bevacizumab re-treatment after front-line bevacizumab-containing therapy seems a reasonable option.

Findings from ENCOURAGE suggest that continued education about the importance of blood pressure monitoring and prompt management of low-grade hypertension remains important in patients receiving bevacizumab. Recently published consensus guidelines developed by cardiologists, medical oncologists, a general practitioner, and specialist oncology nurses, all with experience of treating patients with bevacizumab and/or hypertension, recommend blood pressure measurement in all patients before each bevacizumab infusion and provide clear recommendations for hypertension management (Plummer et al., 2019). They also recommend close communication between a patient’s oncologist and general practitioner and clear specific instructions about target blood pressure and management.

In summary, these results from patients with newly diagnosed ovarian cancer treated with bevacizumab-containing therapy in a real-life setting were relatively consistent with findings from the randomized phase 3 GOG-0218 and ICON7 trials (Burger et al., 2011; Perren et al., 2011). Continued education on blood pressure monitoring and management of hypertension remains important, particularly as the use of bevacizumab with PARP inhibition becomes standard of care.

## Data Availability

The datasets presented in this article are not readily available because currently no mechanism is in place to allow sharing of individual deidentified participant data. Requests to access the datasets should be directed to ARCAGY–GINECO (bvotan@arcagy.org) and will be considered on a case-by-case basis.

## Abbreviations

CI, confidence interval; FIGO, International Federation of Gynecology and Obstetrics; GINECO, Groupe d’Investigateurs Nationaux pour l’Etude des Cancers de l’Ovaire et du sein; INCa, French National Cancer Institute; OS, overall survival; PFS, progression-free survival; PARP, polyADP ribose polymerase.
